# Current Concepts of Biomaterial Scaffolds and Regenerative Therapy for Spinal Cord Injury

**DOI:** 10.3390/ijms24032528

**Published:** 2023-01-28

**Authors:** Hidenori Suzuki, Yasuaki Imajo, Masahiro Funaba, Hiroaki Ikeda, Norihiro Nishida, Takashi Sakai

**Affiliations:** Department of Orthopedics Surgery, Yamaguchi University Graduate School of Medicine, Yamaguchi 755-8505, Japan

**Keywords:** biomaterial, combination therapy, regenerative medicine, scaffold, spinal cord injury

## Abstract

Spinal cord injury (SCI) is a catastrophic condition associated with significant neurological deficit and social and financial burdens. It is currently being managed symptomatically, with no real therapeutic strategies available. In recent years, a number of innovative regenerative strategies have emerged and have been continuously investigated in preclinical research and clinical trials. In the near future, several more are expected to come down the translational pipeline. Among ongoing and completed trials are those reporting the use of biomaterial scaffolds. The advancements in biomaterial technology, combined with stem cell therapy or other regenerative therapy, can now accelerate the progress of promising novel therapeutic strategies from bench to bedside. Various types of approaches to regeneration therapy for SCI have been combined with the use of supportive biomaterial scaffolds as a drug and cell delivery system to facilitate favorable cell–material interactions and the supportive effect of neuroprotection. In this review, we summarize some of the most recent insights of preclinical and clinical studies using biomaterial scaffolds in regenerative therapy for SCI and summarized the biomaterial strategies for treatment with simplified results data. One hundred and sixty-eight articles were selected in the present review, in which we focused on biomaterial scaffolds. We conducted our search of articles using PubMed and Medline, a medical database. We used a combination of “Spinal cord injury” and [“Biomaterial”, or “Scaffold”] as search terms and searched articles published up until 30 April 2022. Successful future therapies will require these biomaterial scaffolds and other synergistic approaches to address the persistent barriers to regeneration, including glial scarring, the loss of a structural framework, and biocompatibility. This database could serve as a benchmark to progress in future clinical trials for SCI using biomaterial scaffolds.

## 1. Introduction

**Figure 1:**
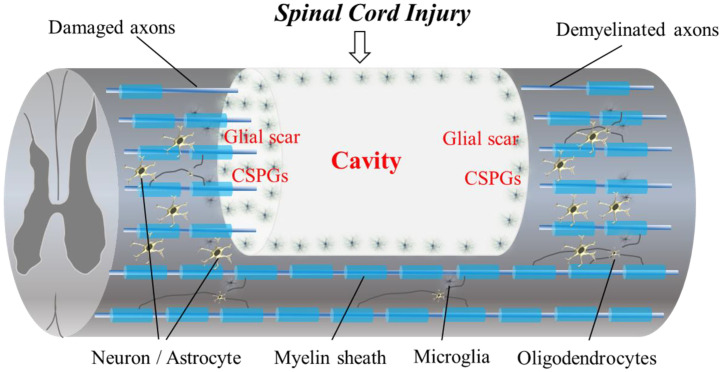
The diagram shows the pathophysiological events in SCI. Progressive demyelination results in the degeneration of axonal fibers. A cavitation occurs in the epicenter. Hypertrophic astrocytes with very long processes over the tips of non-regenerating fibers form a barrier known as a glial wall around the cavitation. In response to injury, microglial cells transform into active phagocytic microglia and exhibit chemotaxis. The presence of CSPGs creates an inhibitory environment for axonal regeneration. In addition, CSPG also inhibits the migration and differentiation of oligodendrocyte progenitor cells.

Spinal cord injuries (SCIs) are a serious problem for those affected. The physical, emotional and economic problems caused by SCI generally considerably limit an individual’s functionality and are a burden on society. One recent survey reported an annual incidence of SCI of approximately 54 cases per one million people [1,2], with an estimated yearly incidence worldwide of 250,000–500,000 cases [3]. The spinal cord has very little ability to spontaneously or functionally regenerate itself, thus resulting in serious and often permanent disabilities. Unfortunately, 95% of patients with SCI are in the chronic phase [4]. The cause of SCI is triggered by several types of physical impacts, including traffic accidents, falls and sports injuries, etc., in which spinal vertebrae, facet joints, disks and ligamentous structures are injured and lose stability. The impact load is therefore transferred to the spinal cord and injures it. The external insult is reflected in primary spinal tissue damage and neural cell death in the acute phase, while a subsequential secondary cascade of degenerative events is started [5] (Figure 1).

Spinal surgery is often necessary to reduce the cervical dislocation and to remove fragments of bones, herniated disks, foreign objects or fractured vertebrae that appear to be compressing the spinal cord and cervical nerve roots. Surgery might also be needed to stabilize the spine to prevent future pain or deformity. Unfortunately, there is no way to reverse damage to the original spinal cord [1,2,3,4,5,6]. 

Recent progress in medicine, biology and biomaterials engineering in neurosurgery, biomaterial development, cell culture and tissue engineering has allowed for new therapies in SCI. This has contributed to the possibility of healing traumatic SCI and preventing further neurodegeneration [5,6,7,8,9,10,11,12,13,14,15,16,17,18,19]. It remains a severe clinical challenge to effectively treat SCI due to the poor regenerative capacity and complex anatomy of the spinal cord. Several biomaterials that act as scaffolds for axonal growth, cells and neurotrophic factors have become excellent candidates to support the regeneration of the spinal cord.

Recent review articles in new treatments for SCI have mentioned the possibility of clinical applications and the progression of a new regenerative therapy for SCI, including the most recent preclinical results and clinical trials [5,6,18,19,20,21,22,23,24,25,26,27,28,29,30,31,32]. However, there have been few review articles in SCI that have mentioned the status of the use of recent biomaterial scaffolds for regenerative therapy and summarized the strategies with simplified results data. In this context, therefore, particular attention has been drawn to biomaterials and nanotechnology-enabled products for the controlled delivery and sustained release of various moieties, including drugs, bioactive molecules and cells [5].

**Figure 2:**
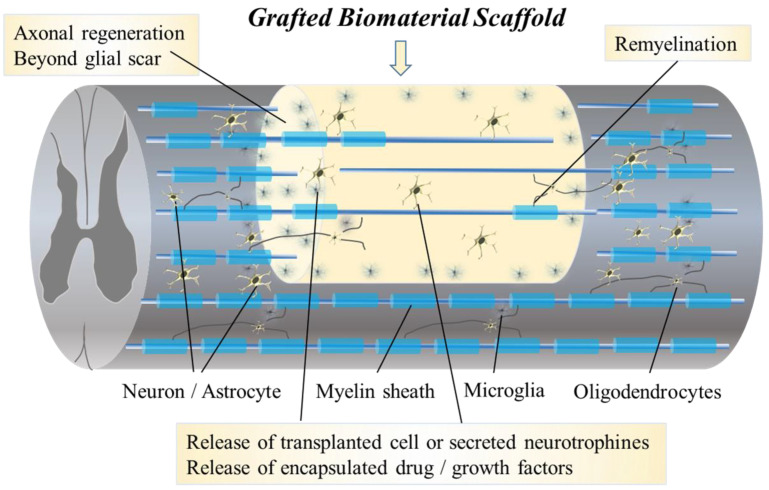
The diagram shows the pathophysiological change following a biomaterial scaffold graft. Certain biomaterials contain stem cells, drugs, neurotrophines or growth factors, etc. Grafted biomaterials support axonal regeneration beyond the glial scar as a scaffold. Grafted biomaterial scaffolds release the transplanted cells or secreted neurotrophines, and, in addition, they release the encapsulated drugs or growth factors, etc. They support the formation of new synaptic circuits and connectivity between host neurons and axons, and, in addition, they improve morphological and behavioral outcomes after experimental SCI. Oligodendrocytes derived from grafted stem cells remyelinate damaged host axons. Regenerated and remyelinated axons pass through the injured lesion and connect to other host neurons supported by interneurons and glial cells derived from grafted stem cells.

Aiming to set a framework for future clinical use, we briefly describe the most recent developments in biomaterial scaffolds for SCI treatment including combination therapy with cell-seeded materials or innovative drug delivery systems. This review article focuses on previously published biomaterial scaffolds applied to encourage spinal cord regeneration following SCI and summarizes the most recent findings from preclinical and clinical studies using biomaterial scaffolds and other combinatory therapy to treat SCI (Figure 2). An overview of SCI is provided, and the current aspects of clinical biomaterial scaffolds therapy are discussed. First, barriers to regeneration and the pathophysiology of SCI are described. Then, the several categories of biomaterial scaffolds applied in regeneration therapy for SCI are compared. We also review and discuss the current concepts of biomaterial scaffolds in combinatory treatment for SCI and chronic SCI. In the last section, we describe the use of biomaterial scaffolds in ongoing clinical trials for SCI.

## 2. Barriers to Regeneration and the Pathophysiology of SCI (Figure 1)

The regeneration of the adult mammalian central nervous system (CNS) and spinal cord is difficult due to its limited plasticity [1,6,14,15,16]. Cavitation occurs in the epicenter of a CNS lesion, with this becoming surrounded by connective scar tissue containing cerebrospinal fluid. Reactive astrocytes transform into scar-forming astrocytes that slow the crossing of regenerating axons into the lesion. Certain inflammatory immune cells also remain around the lesion epicenter at the site of the SCI [1,6,14,15,16,17,18,19]. Following SCI, astrocytes are activated, and they proliferate and migrate to the perilesional region to form processes in a dense interwoven network, depositing chondroitin sulfate proteoglycans (CSPGs) into the extracellular matrix (ECM). Dystrophic axons surround the epicenter of the injury and are trapped in the dense meshwork of scar tissue [15,17]. Biomaterial scaffolds that generate specific microenvironmental cues in a three-dimensional (3D), controlled fashion to enhance the survival, infiltration and differentiation of cells [18] are used for spinal cord regeneration following injury.

## 3. Systematic Review of Biomaterial Scaffolds Applied for SCI

Below, we review the biomaterial scaffolds applied in regeneration therapy for SCI from selected articles following our literature search.

### 3.1. Literature Search and Inclusion Criteria

In conducting our systematic review, we followed the guidelines of PRISMA (Preferred Reporting Items for Systematic Re-views and Meta-Analyses) (https://prisma-statement.org/, accessed on 1 April 2022). From the PubMed/MEDLINE database, we initially identified relevant articles published up until 30 April 2022 that met the search terms “Spinal cord injury”, and [“Biomaterial”, or “Scaffold”]. After reviewing all of the articles’ titles, we chose titles relevant to our review. Articles not written in English were excluded. After reviewing the abstracts of these titles, we excluded those articles with unrelated titles. Then, following a review of the reference lists in the remaining articles, we identified additional relevant publications and added them. Finally, we performed a full-text review of these articles, and those without a full text available or that were in vitro studies were excluded.

The criteria for article selection were: (1) biomaterial scaffolds were used for SCI or the spinal cord transection model; (2) the treatment outcome was described in detail; and (3) the articles were written in English. In this review of biomaterial scaffolds, we focused on the treatment efficacy of biomaterial scaffolds used in regeneration therapy for SCI in in vivo studies.

### 3.2. Study Selection

**Figure 3:**
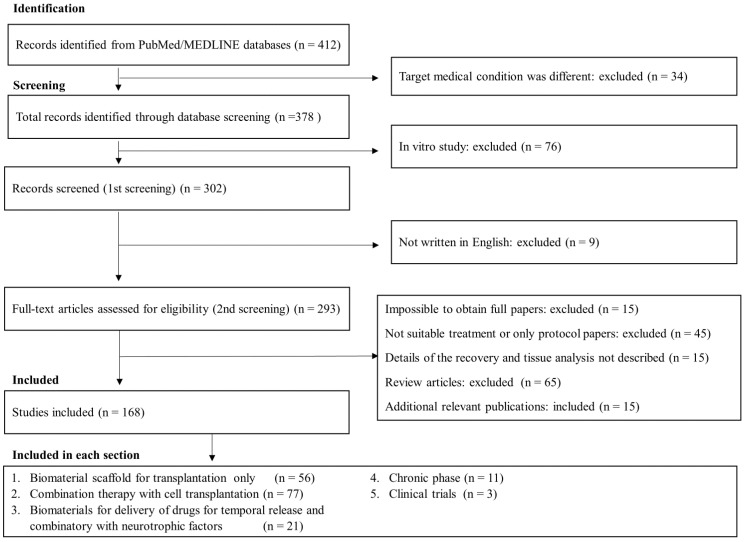
Flowchart of the screening process in this systematic review.

Our database search identified 412 potential articles. A review of the titles and a removal of duplicates resulted in the exclusion of 110 articles, leaving 302 articles for abstract and full-text review, after which 140 articles were excluded. The reasons for study exclusion were: (1) review articles; (2) only protocol papers; (3) treatment outcomes were not described in detail; (4) not suitable after discussion; and (5) not blind studies. After the inclusion of 15 additional relevant publications, 168 studies met the criteria for review. The search flow diagram is depicted in Figure 3. Two reviewers (H.S. and Y.I.) independently screened the titles and abstracts of the studies identified by the search strategy to determine their potential relevance. The full texts of these potentially relevant studies were retrieved, and these same reviewers evaluated them for eligibility. Disagreements were resolved via consensus, and a third independent reviewer (T.S.) resolved any disputes if consensus could not be reached.

### 3.3. Assessment of Quality and Risk of Bias

Two review authors (H.S. and M.F.) independently assessed the studies for risk for bias using the Cochrane Back Review Group “risk of bias” tool, and a third reviewer (H.I.) and another author (N.N.) helped to resolve any disagreements. All included studies were in vivo animal studies. Therefore, there was no risk of bias in regard to the diagnostic criteria, validity and reliability of the measurements, and no studies had selection bias. In addition, the number of analyzed animals was statistically acceptable in all studies, and they reported the random selection of the control and the scaffold treatment groups.

As shown in tables 168 articles were selected in the present review.

## 4. Categories of Biomaterial Scaffolds Applied in Regeneration Therapy for SCI

The biomaterial scaffolds used in spinal cord regeneration can be classified according to the required structure and physical and biological properties of the prospective tissue construct applied in SCI. The categories of the biomaterial scaffolds used in spinal cord regeneration include hydrogels, biodegradable scaffolds, the use of micro/nanofibers as instructive biomaterials and drug-delivering biomaterials [29,30,31,32].

### 4.1. Hydrogels

Hydrogels are one of the most appealing and frequently engineered scaffolds. They are made up of 3D cross-linked biocompatible polymeric macroporous networks that supply the permissive microenvironment and guidance cues necessary for axonal growth [7,33,34,35,36,37,38,39,40,41,42,43,44,45,46,47,48,49,50,51,52,53,54,55,56,57,58,59,60,61,62]. The hydrogel scaffolds used alone in studies applied for SCI treatment are shown in Table 1 [33,34,35,36,37,38,39,40,41,42,43,44,45,46,47,48,49,50,51,52,53,54,55,56,57,58,59,60,61,62]. Hydrogels are hydrated networks that mimic the ECM of soft tissues [30,31]. Natural hydrogels usually contain fibrillar proteins within a hydrated glycosaminoglycan network that can enhance cell adhesion and migration in the lesion site. The natural polymers used for nerve tissue engineering include agarose, alginate, chitosan, collagen, fibrin, fibronectin, hyaluronic acid (HA) and Matrigel™ [30,31]. Natural polymers deliver excellent biomimicking, but synthetic hydrogels have also attracted attention because they can potentially control their rate of degradation and for their mechanical properties [30,31].

We reviewed the effects of hydrogel scaffolds on pathophysiologiocal events and motor functional recovery (Table 1) [33,34,35,36,37,38,39,40,41,42,43,44,45,46,47,48,49,50,51,52,53,54,55,56,57,58,59,60,61,62]. Several types of hydrogels have been reported to date [33,34,35,36,37,38,39,40,41,42,43,44,45,46,47,48,49,50,51,52,53,54,55,56,57,58,59,60,61,62]. Biopolymer-based hydrogel scaffolds are categorized into natural polymers, synthetic polymers and self-assembling peptides according to the origin of the biomaterial used [7,63]. Twenty-nine articles revealed axonal growth into an implanted biomaterial scaffold [33,34,35,36,37,38,39,40,41,42,43,44,45,46,47,48,49,50,51,52,53,54,55,56,57,58,60,61,62], and thirteen papers showed motor functional recovery following scaffold implant in in vivo studies [33,36,37,38,43,48,50,53,56,57,58,60]. Several articles revealed an anti-inflammatory effect [38,46,48,50,54,59] and angiogenesis [45,54,55,56,57,58] following the implantation of the biomaterial scaffold in the spinal cord (Table 1).

### 4.2. Biodegradable Scaffolds

The biodegradable polymers currently used in devices approved by the US Food and Drug Administration provide attractive building blocks for synthetic tissue scaffolds because their biocompatibility has already been established and the regulatory approval process is simple. The biodegradable scaffolds used to treat SCI can be combined with hydrogels. Among the most widely used biodegradable polymers are hydrophobic polyesters such as poly (lactic acid) (PLA), poly (lacticco-glycolic acid) (PLGA) and poly (ε-caprolactone) (PCL). These polymers have been used in sutures and resorbable orthopedic fixation devices because their synthetic fibers provide good mechanical properties and adjustability [53,54]. PLA is a biocompatible lactic acid polymer. The neatly arranged PLA microfibers in transplants promoted the regeneration of CNS tissues [64]. As a product of the reaction between PGA and PLA, which are biodegradable and synthetic polymers, PLGA co-polymer scaffolds show good porosity, hydrophilicity and biodegradability and are usable as drug carriers. One drug delivery device takes the form of a PLGA-based nerve conduit used to control the local delivery of nerve growth factor (NGF) and is applied at the site of the peripheral nerve gap injury [64]. Biocompatible and biodegradable aliphatic polyester make up PCL scaffolds, and this polyester has been used widely in many biomedical applications including bioactive drug delivery for spinal cord regeneration. Other important biomaterials used in SCI include chitosan and gelatin [64]. These are frequently implanted surgically into lesions and are synthesized via electrospinning techniques to decrease organic solvent use [64]. QL6, a biodegradable peptide which self-assembles into nanofiber scaffolds when injected into the spinal cord cavity, has been shown to reduce apoptosis, inflammation and astrogliosis, leading to electrophysiological and behavioral improvements [7,65]. Furthermore, when co-transplanted with NPCs, QL6 enhanced graft survival and promoted differentiation towards neuronal and oligodendroglial cell fates [7,65]. In another type of biodegradable scaffold, functional sequence SIKVAV-modified PA hydrogels implanted into a rat model of SCI improved histological and functional recovery [66].

We reviewed the effects of biodegradable scaffolds on pathophysiologiocal events and motor functional recovery when applied for SCI treatment (Table 2) [66,67,68,69,70,71,72,73,74,75,76,77,78,79,80,81,82,83,84,85,86]. Most articles revealed axonal growth into implanted biodegradable scaffolds [66,67,68,69,70,71,72,73,74,75,77,79,81,82,83,84,85,86]. Seven papers showed motor functional recovery following scaffold implantation in in vivo studies [69,71,79,81,83,84,85]. Several articles revealed an anti-inflammatory effect [81,84,85] and angiogenesis [66,71,72,82,84,85] following the implantation of the biodegradable scaffold in the spinal cord (Table 2).

### 4.3. Nano- and Micro-Scale Scaffolds as Instructive Biomaterials for SCI 

The recent development of various nanomaterials is offering promising new ways to treat SCI by crossing the blood–spinal cord barrier to deliver therapeutics. Several articles revealed the development of nanomaterials that can modulate inflammatory signals, target inhibitory factors within a lesion and promote axonal regeneration following SCI [87,88,89,90,91,92,93,94,95,96,97,98,99,100,101,102,103,104].

Experimental models for SCI treatment are increasingly being used to study nanoparticles. The extremely diverse composition of nanoparticles includes polymers, metals and metal oxides, silica and biological molecules [87]. The biocompatibility of polymeric nanoparticles has allowed them to become the most extensively used means of delivering drugs to the spinal cord. Unlike with drugs, topographical cues in the implanted scaffolds at the lesion site can physically guide the extension of new axons [87,88,89,90,91]. The electrospinning of nanofibers is advantageous because it permits the production of highly porous 3D scaffolds with a large surface area that aids in cell adhesion [87]. Spontaneous self-assembling peptides can also form nanofibrous hydrogels that are composed of natural amino acid sequences, rendering them nonimmunogenic, nontoxic and biodegradable [86,87]. Self-assembling peptides have an additional advantage in that they can undergo gelation in physiological conditions, and their morphology mimics in vivo ECM [7,91]. The ionic complementarity of many common self-assembling peptides allows them to form nanofibrous structures. Several articles reported using other materials for nanoscale scaffolds [87,88,89]. Because of their size, which closely mimics that of ECM proteins, and their high surface area, carbon nanotube nanostructures have shown promising effects in neural regeneration applications. Electrospinning produces micro- and nanofibers that can simulate collagen fibers in the ECM [88]. RADA16-I hydrogels were used in an experimental SCI model, which proved that self-assembling peptide hydrogels could promote recovery from SCI [91]. Further development produced functionalized RADA16-I hydrogels with a bone marrow-homing motif (BMHP1) [91,99]. These researchers inserted a 4-glycine-spacer into the hydrogels to facilitate scaffold stability and expose more bi motifs. Their results showed that RADA16-I hydrogels can increase cell infiltration, basement membrane deposition and axon regeneration in SCI [104].

These kinds of nanoscale scaffolds and nanofibers were mainly used for drug delivery systems (DDSs) (refer to Section 5). Therefore, there were only a small number of studies on nanoscale scaffolds applied to SCI treatment [89,90,91,92,93,94,95,96,97,98,99,100,101,102,103,104]. We reviewed the effects of nanoscale or microscale biomaterial scaffolds on pathophysiological events and motor functional recovery (Table 3) [89,90,91,92,93,94,95,96,97,98,99,100,101,102,103].

## 5. Biomaterial Scaffolds in Combinatory Treatment Used for DDSs in SCI Treatment

Hydrogels and biodegradable and nanomaterial scaffolds were also widely used for DDSs as a combinatory treatment for SCI. Scaffolds provide a surrounding 3D environment that promotes the in vivo adhesion, migration and differentiation of cells [104]. In SCIs, the scaffolds, as a matrix for cell, drug and other bioactive molecule delivery, bridge the SC lesion cavity to structurally and chemically support axonal regrowth and stimulate the regeneration of host tissue [104]. Several concepts of SCI treatment using biomaterial scaffolds containing basic fibroblast growth factor, methylprednisolone, calcium responsive composite, neurotrophic factors, anti-Nogo and anti-inflammatory agents have been reported [105,106,107,108,109,110,111]. Strategies for SCI repair are still limited in part by poor drug delivery techniques. However, several ideal DDSs using degradable/nondegradable biomaterial scaffolds have been developed that can provide the localized release of growth factors or other neuroprotective agents from an injectable gel form [112,113,114]. We reviewed the studies of biomaterial scaffolds used in combinatory treatment as DDSs for SCI that are shown in Table 4 [105,106,108,109,110,114,115,116,117,118,119,120,121,122,123,124,125,126,127,128,129,130].

Gelatin hydrogel containing basic fibroblast growth factor that was injected into a rat model of SCI showed better performance in relieving mechanical allodynia [105]. Biomaterials containing methylprednisolone can also enhance axonal regeneration and reduce inflammation [106]. The exceptional ability of alginate/chitosan/genipin hydrogels, which show a high sensitivity to Ca2+ composites, to regulate astrocyte behavior and prevent Ca^2+^-related secondary neuron damage during acute SCI was shown in an in vitro study [107]. A significant therapeutic role was shown for the local delivery of constitutively active Rho GTPases, Cdc42 and Rac1 with the microtubule-mediated slow release of brain-derived neurotrophic factor (BDNF) in overcoming CSPG-mediated failure of regeneration following SCI [108]. A previous article reported on HA hydrogels that were developed to blend with the anti-Nogo receptor antibody (antiNgR). Hydrogel combinations with PLGA microspheres containing BDNF and vascular endothelial growth factor (VEGF) were also reported [109]. Following the implantation of a composite modified by binding with an antiNgR and further mixing with PLGA microspheres containing BDNF and VEGF into an injured area created by the dorsal hemisection of the spinal cord at T9–10 in rats, favorable effects were observed that indicated the promotion of spinal repair, including the integration of the implants with host tissue, the inhibition of inflammation and gliosis [99]. The implantation of bFGF combined with hydroxyl ethyl methacrylate [2-(methacryloyloxy) ethyl] trimethylammonium chloride (HEMA-MOETACL) hydrogels resulted in the promotion of nerve tissue regeneration and functional recovery using hydrogels in a SCI model [110]. These results also suggest the importance of the proper matching of the functional sequence and hydrogels in the synthesis of functional hydrogels. The combination of collagen–laminin scaffolds with 5-NOT treatment also promoted axonal regrowth at the site of SCI as indicated by the expression of NF200 and monoaminergic and glutamatergic reinnervation [113].

Several other combinatory approaches were reported that combined biomaterial scaffolds with rehabilitation and the release from the scaffolds of neurotrophin 3 factor (NT-3), Nogo-66 receptor antibody, ibuprofen/triiodothyronine, sonic hedgehog/retinoic acid, dibutyryl cyclic adenosine monophosphate and rho-A-kinase inhibitor [109,114,115,116,117,118,119,120,121,122,123,124,125]. Other scaffolds, such as silk fibroin combined with neurotrophic factors [125,126], fibrin scaffolds containing growth factors [127] and the polycistronic delivery of IL-10 and NT-3 [128], showed desirable therapeutic potential in terms of SCI treatment. These therapies promoted the differentiation, proliferation and viability of transplanted cells.

The effects of biomaterial scaffolds in combinatory treatments as DDSs applied for SCI treatment on pathophysiological events and motor functional recovery data are summarized in Table 4.

## 6. Biomaterial Scaffolds in Combinatory Treatment with Cell Therapy for SCI

As an appealing therapeutic approach for SCI, cell therapy can provide significant neuroprotection, the recovery of functionaility through cell replacement, trophic support and the modulation of immune factors [18,131], and, thus, clinical trials have also been started in humans [132,133]. As mentioned above, we noted the use of biomaterials for SCI repair because of the structural or active growth support they provide to damaged axons. Furthermore, biomaterials have the ability to function as cell delivery platforms for cells and therapeutic molecules and as a local depot for sustained drug release. Both cell regeneration and tissue reconstruction can be achieved when these two therapeutic methods are combined. By following the basic operating principle of this modality, i.e., the combination of exogenous cells and scaffolds to form live scaffolds, we can expect the synergic effects of stem cells and scaffolds to occur. These live scaffolds can be implanted into animals through injection or surgical implantation without side effects [104]. We reviewed the preclinical studies using biomaterial scaffolds in combination with cell therapy, so called multipotent stem cells, for the treatment of SCI. The combinatory treatments with cell therapy are summarized in Table 5 [36,117,134,135,136,137,138,139,140,141,142,143,144,145,146,147,148,149,150,151,152,153,154,155,156,157,158,159,160,161,162,163,164,165,166,167,168,169,170,171,172,173,174,175,176,177,178,179,180,181,182,183,184,185,186,187,188,189,190,191,192,193,194,195,196,197,198,199,200,201,202,203,204,205,206,207,208,209,210,211,212,213,214,215,216,217,218,219,220,221,222,223,224,225,226,227].

### 6.1. Exogenous Neural Stem/Progenitor Cells and Biomaterial Scaffolds

Therapies using exogenous neural stem/progenitor cells (NPCs) show particular promise because these cells can potentially differentiate into all three neuroglial lineages—neurons, astrocytes and oligodendrocytes—to regenerate neural circuits, remyelinate denuded axons and provide trophic support to endogenous cells [15,18,19,131]. However, the transplantation of NPCs, especially in the chronic phase, showed several issues regarding tissue regeneration in terms of the survival rate of NPCs and insufficient integration with injured spinal cord [15,18]. Many researchers have tried novel combinatorial treatments with biomaterial scaffolds and NPCs, and several articles have reported the expected synergic effects of these grafts [15,65]. Over the previous decade, when NSCs were delivered via a supporting scaffold matrix, significant outcomes regarding functional recovery were consistently observed in the preclinical stage [6,7,9,19,65], but these favorable results have yet to be translated into clinical use. In the meantime, clinical developments that affect the safety and feasibility of implantable biomaterials for CNS repair are currently underway. The safety and feasibility of the transplantation of the NeuroRegen implantable collagen scaffold in completely chronic patients with SCI has been reported, although the sample size in these studies is small [132,133].

NPCs derived from induced pluripotent stem cells (iPSCs), embryonic stem cells or brain or spinal cord within biomaterial scaffolds are also being used for the treatment of SCI [36,134,135,136,137,138,139,140,141,142,143,144,145,146,147,148,149,150,151,152,153,154,155,156,157,158,159,160,161,162,163,164,165,166,167,168,169,170,171,172,173,174,175,176,177] (Table 5). SCI rats receiving the transplantation of NPCs in Matrigel showed improvements in behavioral recovery and the expression levels of neuronal and reactive astrocyte markers [162]. A fabricated biodegradable hybrid inorganic scaffold comprised of biodegradable MnO_2_ nanosheets enhanced the attachment and differentiation of iPSC-derived NSCs in the site of SCI [134]. Fibrin scaffolds and stem cell therapy designed to immobilize cells and release growth factors (NT3, glial-derived neurotrophic factor [GDNF] and platelet-derived growth factor-A [PDGF]) from fibrin achieved better recovery from SCI [158,159]. NPCs used with self-assembling peptide QL6 decreased the formation of cystic cavities and inflammation and enhanced synaptic connections through a reduction in astrogliosis and CSPG, thus improving forelimb functionality in an SCI model of cervical injury [7,65]. Laminin-coated hydrogel enhanced iPSC-NPC viability and promoted host axon and astrocyte growth in lesion sites [152]. Another article reported the manufacture of NPCs biased toward an oligodendrogenic fate and the upgrading of the ChABC delivery system via a crosslinked methylcellulose biomaterial. This combinatorial therapy resulted in the promotion of oligodendrocyte differentiation, remyelination and synaptic connectivity [165]. A linearly ordered collagen scaffold modified with N-cadherin promoted the migration and differentiation of endogenous neural/progenitor stem cells, which produced a desirable therapeutic effect in rats following SCI [36]. One treatment showing great potential for SCI treatment was the combination of a collagen microchannel scaffold and paclitaxel liposome, which induced the neuronal differentiation of NSCs and neuron and axon growth [144]. A different group reported the benefits of combining NPCs and K2(QL)6K2 (QL6), an aqueous self-assembling peptide that aggregates into a stable nanofiber gel due to multiple non-covalent interactions [7,103]. In a study exploring the modification of a scaffold with PDGF-A to induce oligodendrocyte differentiation, NPCs cultured in a hydrogel blend of hyaluronan and methylcellulose (HAMC) modified with PDGF-A showed improved survival and the higher differentiation of cells into oligodendrocytes. SCI rats transplanted with NPCs cultured in this hydrogel blend showed reduced cavitation, improved graft survival with increased differentiation of oligodendrocytes and improved behavioral recovery [170]. These researchers further modified the HAMC-PDGF-A scaffold with arginine-glycine-aspartic acid (RGD) peptide to improve the engraftment and survival of human iPSC-derived oligodendrocyte precursor cells (OPCs). iPS cell-derived OPCs transplanted in HAMC-RGD/PDGF-A had higher rates of survival and engraftment than iPS cell-derived OPCs transplanted with media did [171].

### 6.2. Nanoscaffolds and Stem Cell Grafts

Neuroinflammatory agents such as metalloproteinase and neurotoxic cytokines that are secreted after CNS injuries can lead to a reduction in the neuroinhibitory microenvironment in the region of injury [177]. Drug-loaded 3D nanoscaffolds designed to reduce neuroinflammatory agents were fabricated using a layer-by-layer method in which chitosan polymer functionalized manganese oxide nanosheets for fabrication into a 3D esoporous structure. Methylprednisolone and laminin were also added as cell-adherent ECM ligands to the fabricated scaffold, and its effects on SCI treatment were evaluated with iPSCs. This study confirmed functional recovery and axonal growth due to stem cell differentiation and the suppression of fibrotic scar fabrication in an in vivo model of SCI [177,178]. By mimicking the ECM, the fibrous structure of the nanofibrous scaffolds provided an ideal platform for the attachment, proliferation and differentiation of stem cells [178]. The capability of multichannel nanofibrous scaffolds using poly-L-lysine integrated NT-3 to promote the recruitment and differentiation of endogenous NPCs facilitated synapse formation and enhanced locomotor recovery, thus promoting the treatment of SCI [166].

### 6.3. Mescenchymal Stem Cells (MSCs) and Biomaterial Scaffolds: Bone Marrow MSCs, Umbilical MSCs, Wharton’s Jelly-Derived MSCs and Adipose-Derived MSCs

MSCs are a type of stem cell present in adults that can differentiate into mesodermal-derived tissues such as bone, cartilage, blood vessels and cardiomyocytes. MSCs used for clinical purposes are derived from tissues such as bone marrow, umbilical cord and cord blood and fat. MSCs have important biological activities for tissue repair, such as anti-inflammatory effects, growth factor secretion and the promotion of angiogenesis in addition to having a low risk of tumor formation. Moreover, MSCs exhibit remarkable autocrine and paracrine activity. MSCs can secrete various soluble molecules that exert anti-inflammatory potential, including tumor necrosis factor (TNF)-β1, interleukin (IL)-13, IL-18 binding protein, ciliary neurotrophic factor (CNTF), NT-3, IL-10-, IL-12p70, IL-17E and IL-27 [179]. Furthermore, the release of pro-inflammatory cytokines such as interferon, TNF and IL-10 can also be inhibited by MSCs to modulate cytokine production in the host. These cells also produce a wide variety of growth-promoting molecules, including BDNF, CNTF, GDNF, leukemia inhibitory factor, NGF and neurotrophin 3 (NT-3) and ECM proteins such as laminin, fibronectin and collagen I/III and IV [176]. One essential method used by MSCs to secrete biological factors is through extracellular vesicles, which include microvesicles and exosomes. [179]. The co-transplantation of biomaterial and MSCs that have been manipulated or genetically edited to express certain proteins causes neuroprotective and anti-inflammatory effects that induce anti-inflammatory mechanisms [180]. The transplantation of biomaterial-supported MSCs lessens fibrosis during the early process of secondary SCI and further attenuates secondary glial scarring [181]. Biomaterial-supported MSCs that were transplanted into the damaged region subsequently prevented the accumulation of CSPGs, which make up the glial scar, and significantly promoted the myelination of axon fibers and synapse formation [181]. MSCs can cooperate with biomaterials to support the growth of stem cells and endogenous neuronal cells by bridging the gap. A nanofibrous scaffold of polypyrrole/polylactic acid was also used as a platform to deliver bone marrow mesenchymal stem cells (BMSCs) to the site of SCI. BMSCs are beneficial cells with the ability to differentiate into different neural cell types and appear to be proper candidates for replacing damaged cells in SCI. Furthermore, these cells secrete neurotrophic factors to protect the injured spinal cord [182]. The application of this formulation promoted myelination and axon regeneration, enhanced the microenvironment at the site of injury and synergically reduced neuronal apoptosis at the injury site in the spinal cord [182].

The combination of Matrigel and neural-induced adipose-derived MSCs reduced fibrosis from secondary injury processes and improved neuronal regeneration [182]. According to a behavioral and electrophysiological analysis, 3D-printed collagen/silk fibrin scaffolds carrying umbilical secretomes of MSCs improved hindlimb locomotor functionality [183]. Wharton’s jelly-derived MSCs applied with integrin-binding peptide RGD bridged the lesion cavity, supported vascularization, upregulated related gene expressions and increased axonal sprouting into the lesion [184]. The transplantation of human umbilical cord MSCs seeded in collagen scaffolds also reduced scar formation and promoted functional recovery in chronic SCI [183,185].

The combination therapies of biomaterial scaffolds and MCSs for SCI are summarized in Table 5 [181,182,183,184,185,186,187,188,189,190,191,192,193,194,195,196,197,198,199,200,201,202,203,204,205,217]. Several articles used combinations with cells other than NPCs, i.e., Schwann cells (SCs) and/or olfactory ensheathing cells, to support the survival, integration and migration of grafted cells [163,218,219,224].

### 6.4. Schwann Cells, Olfactory Ensheathing Cells, Astrocytes and Other Cell Grafts and Biomaterial Scaffolds

SCs are neuroglial cells that drive axon regeneration and myelination in the peripheral nervous system, but they also perform an analogous function when transplanted into the spinal cord. Furthermore, SCs can be isolated from a patient’s own nerves and expanded in vitro prior to implantation, making them an exceptional cell type for autotransplantation therapy in SCI [216]. Numerous preclinical studies have established the functionality of SCs in transplantation [211]. In this section, we review the studies on the combination of biomaterial scaffolds with SCs [210,211,212,213,214,215,216,217,218,219,220,221,222,223,224] shown in Table 5.

One study showed that long-distance regeneration could occur from CNS neurons that project through a scaffold construct into distal tissue implanted with biodegradable PLGA scaffolds loaded with SCs [154]. Other studies found that a poly-b-hydroxybutyrate scaffold, positively charged oligo[poly(ethylene glycol) fumarate] (OPF+) or resorbable poly(-hydroxyacid) guidance channels containing SCs promoted the attachment, proliferation and survival of grafted cells and supported marked axonal regeneration within the graft [210,211,212,213,214,215,216,217,218,219].

The efficacy of other combinatory cell sources on SCI treatment was also reported. The transplantation into an SCI rat model of dental pulp stem cells combined with chitosan scaffolds resulted in the marked recovery of hind limb locomotor functions by increasing the levels of BDNF, GDNF, beta-NGF and NT-3 [220]. The support of the spinal cord structure and induction of cell/tissue polarity were also achieved by the injection of dental follicle cells combined with aligned PCL/PLGA electrospun material [221]. In glial scars, astrocytes have been shown to be important for spontaneous recovery from SCI. One paper reported the effect of implanting HA hydrogels containing ECM harvested from embryonic stem cell-derived astrocytes on histologic outcomes following SCI in rats. Protoplasmic embryonic stem-derived astrocyte ECM also showed the potential to treat SCI injury [222]. PLGA complexes inoculated with olfactory ensheathing cells improved the recovery of locomotor functionality in rat models with transected SCI, most likely because these complexes are conducive to a relatively benevolent microenvironment, offer nerve-protective effects and have the ability to enhance remyelination via the promotion of cell differentiation and the inhibition of astrocyte formation [223,224].

Several articles reported on the co-transplantation of several stem cell types and scaffolds [151,153,155,163,218,219,224]. The effect provided by the co-transplantation of NPCs, SCs and PLGA resulted in better behavioral recovery than that from transplantation with NPCs/PLGA alone [151,153,155]. Axonal regeneration and functional recovery in rat SCI were improved after use of a multichannel polymer scaffold seeded with activated SCs and BMSCs rather than by single treatment with each cell type [219].

## 7. Biomaterial Scaffolds in Regeneration Therapy for Chronic SCI

Several combinatory treatments for chronic SCI using stem cells and biomaterial scaffolds were recently reported clinically and in rodent models [36,132,134,202,206,225,226,227,228,229,230,231,232,233,234,235,236]. The creation of an artificial scaffold that mimics the ECM and supports nervous system regeneration remains one of the greatest challenges in regeneration following chronic SCI.

One effective measure to repair chronic SCI is the removal of scar tissue combined with biomaterial implantation [44]. One article revealed that following scar tissue removal in chronic SCI, the implantation of a Taxol-modified linear-ordered collagen scaffold (LOCS + Taxol) could promote axonal regeneration, neurogenesis and electrophysiological and functional recovery [44]. Pivotal features of neural repair were also shown following treatment with reduced graphene oxide scaffolds at 4 months after SCI [224]. These results indicate that even if a patient is in the chronic phase of SCI, the potential for axonal regeneration, neurogenesis and functional recovery are still preserved at the site of the SCI.

The use of other scaffolds to bridge defects was reported in experimental models of chronic SCI [172,225,227,229,230,235,236]. Following the removal of scarring, anisotropic alginate hydrogel scaffolds promoted axonal growth across chronic transections of the spinal cord [234]. Engraftment with this scaffold significantly improved electrophysiological conductivity and locomotor functionality. Scar formation was reduced and functional recovery in chronic SCI was promoted following the transplantation of human umbilical cord-derived MSCs seeded in collagen scaffolds [206,225]. Other articles revealed the efficacy of laminin-coated pHEMA-MOETACl hydrogel [152], HPMA-RGD hydrogels [67] and chimeric self-assembling nanofiber [227,232], but these were combined with iPSC-derived NPCs or MSCs. The treatment of chronic SCI with 3D-aligned nanofiber-hydrogel scaffolds [43,92,229], self-assembling scaffolds, Taxol-modified collagen scaffolds [44], graphene oxide scaffolds [99,235] and nanostructured composite scaffolds [229] were also reported. These articles showed that it is possible to recreate an anatomical, structural and histological framework that can allow for replacement of large hollow tissue gaps in chronically injured spinal cord and encourage axonal regeneration and neurological recovery.

As indicated by many researchers, a multi-disciplinary approach is required to solve the problem of repairing chronic SCI. From this point of view, combinatory treatment using stem cells and biological scaffolds will be an important approach in the treatment of chronic SCI in the future [18,19].

## 8. Biomaterial Scaffolds in Clinical Trials for SCI

In this section, we review the published and ongoing clinical trials of biomaterial scaffolds for SCI (Table 6) (https://www.clinicaltrials.gov/, accessed on 1 August 2022).

One group reported the results of the NeuroRegen clinical trial using the same protocol as that described in Section 6 [132,133,226,233,236,237]. They revealed that the following primary efficacy outcomes of combinatory therapy with BMSCs or MSCs were observed in some patients with chronic SCI: an expansion of the sensation level and motor-evoked potential (MEP)-responsive area, increased activity in the fingers, an enhancement in trunk stability, the return of the sensation of defecation and the recovery of autonomic neural functionality [132,133,226].

Another group provided the result of bridging defects in chronic SCI in a clinical trial using a combination of peripheral nerve grafts and a chitosan–laminin scaffold. Treatment with this combination enhanced regeneration through co-transplantation with bone-marrow-derived MSCs [237]. The grade on the impairment scale of the American Spinal Impairment Association (ASIA) improved from A to C in 12 patients and from A to B in 2 patients [226].

In the phase 2 NCT02688049 clinical study which begun in January 2016, patients with chronic SCI (ASIA grade A) are receiving a NeuroRegen scaffold transplanted with 10 million NSCs after localized scarring is cleared, and after the surgery patients undergo comprehensive rehabilitation combined with psychological and nutritional measures. Ongoing clinical trials of the NeuroRegen Scaffold with the transplantation of BMSCs or MSCs are also being performed in phase 1 and 2 (NCT02352077, NCT02688062) trials. Other clinical studies are ongoing using collagen scaffolds, the RMx Biomatrix or the transplantation of the poly(lactic-co-glycolic acid)-b-poly(L-lysine) scaffold (NCT02510365, NCT03966794, NCT02326662, NCT03762655, NCT02138110), as shown in Table 6.

## 9. Conclusions and Outlook

In this review, we summarized the most recent insights of the preclinical and clinical studies using biomaterial scaffolds in regenerative therapy for SCI and summarized the biomaterial strategies for treatment with simplified results data. One hundred and sixty-eight articles were selected in the present review, in which we focused on biomaterial scaffolds. We separately summarized the preclinical experimental results for hydrogels, biodegradable scaffolds, nano-/microscale scaffolds, biomaterial scaffolds in combinatory treatment used for DDSs, combinatory with cell therapy and regeneration therapy for chronic SCI. In addition, in the last section, we also reviewed ongoing and the most recently completed clinical trials using biomaterial scaffolds for SCI. Presently, a number of clinical and experimental studies have reported positive results showing motor functional improvement, anti-inflammation, scar/cavity reduction, axon growth and angiogenesis promotion in SCI with the use of biomaterial scaffold grafts. Although some inherent limitations still exist in performing human SCI trials, in that animal experiments cannot be directly applied to humans, much basic research and many clinical trials of biomaterial scaffold therapy have already been performed that show promising results. This database could serve as a benchmark for progress in future clinical trials for SCI with biomaterial scaffolds. Nevertheless, we strongly believe that in the near future, biomaterial scaffolds will deliver the radical treatment required to treat patients with SCI.

## Figures and Tables

**Table 1:** Summary of the included studies and the effect of hydrogel application in SCI.

					Effect on Pathophysiological Events				
Author, Year	Location of Injury	Species	Application	Hydrogel (Character)	Anti-Inflammation	Scar/Cavity	Axon Growth	Angiogenesis	Motor Functional Recovery
Sun Y, et al., 2019 [33]	Thoracic	Rat	Implant	Collagen/Chitosan (3D printing)	NA	+	+	NA	+
Marchand R, et al., 1990 [34]	Thoracic	Rat	Implant	Collagen (Self assembling)	NA	+	+	NA	NA
Khan T, et al., 1991 [35]	Thoracic	Rat	Implant	Carbon (Filament)	NA	+	+	NA	NA
Liu W, et al., 2020 [36]	Thoracic	Rat	Implant	Collagen (modified with N-cadherin)	NA	+	+	NA	+
Fan C, et al., 2017 [37]	Thoracic	Rat	Implant	Collagen (binding with EGFR antibody Fab fragment)	NA	+	+	NA	+
Yang B, et al., 2017 [38]	Thoracic	Rat	Implant	Agarose/gelatin/polypyrrole (similar conductivity as the spinal cord)	+	+	+	NA	+
Martín-López E, et al., 2013 [39]	Thoracic	Rat	Implant	Agarose with κ-carrageenan, gelatin, xanthan gum and polysulfone	NA	+	+	NA	NA
Gros T, et al., 2010 [40]	Cervical	Rat	Implant	Agarose	NA	+	+	NA	NA
Kataoka K, et al., 2004 [41]	Thoracic	Rat	Implant	Alginate (Freeze-dried sponge )	NA	+	+	NA	NA
Prang P, et al., 2006 [42]	Cervical	Rat	Implant	Alginate (Anisotropic capillary)	NA	+	+	NA	NA
Cao Z, et al., 2020 [43]	Lumbar	Canine	Implant	Fibrin (Hierarchically aligned fibrin hydrogel)	NA	+	+	NA	+
Yin W, et al., 2021 [44]	Thoracic	Canine	Implant	Collagen (Taxol-modified linear-ordered scaffold)	NA	+	+	NA	NA
Altinova H, et al., 2020 [45]	Cervical	Rat	Implant	Collagen	NA	+	+	+	NA
Gholami M, et al., 2021 [46]	Thoracic	Rat	Implant	Chitosan/alginate/erythropoietin	+	+	+	NA	NA
Stokols S, et al., 2006 [47]	Cervical	Rat	Implant	Alginate (recombinant BDNF protein)	NA	+	+	NA	NA
Zhang Z, et al., 2017 [48]	Lumbar	Canine	Implant	Biomaterial-aligned fibrin	+	+	+	NA	+
Fukushima K, et al., 2008 [49]	Thoracic	Rat	Implant	Collagen (Honeycomb)	NA	NA	+	NA	NA
Zhao X, et al., 2022 [50]	Thoracic	Rat	Implant	Gelatin/hyaluronic acid	+	+	+	NA	+
King VR, et al., 2010 [51]	Thoracic	Rat	Implant	Collagen (viscous fibronectin gel)	NA	NA	+	NA	NA
Cheng H, et al., 2007 [52]	Thoracic	Rat	Implant	Chitosan	NA	+	+	NA	-
Han S, et al., 2018 [53]	Thoracic	Rat	Implant	Agarose (Matrigel)	NA	NA	+	NA	+
Bakshi A, et al., 2004 [54]	Cervical	Rat	Implant	Nonbiodegradable hydrogel (pPHEMA)	+	+	+	+	NA
Zhai H, et al., 2020 [55]	Thoracic	Rat	Implant	ADA16 peptide hydrogel	NA	+	+	+	NA
Hejčl A, et al., 2018 [56]	Thoracic	Rat	Implant	3 Methacrylate hydrogel	NA	+	+	+	+
Zhang Q, et al., 2016 [57]	Thoracic	Rat	Implant	Silk protein/laminin	NA	+	+	+	+
Chai Y, et al., 2022 [58]	Thoracic	Rat	Implant	Bioactive isoleucine-lysine-valine-alanine-valine	NA	+	+	+	+
Yang Y, et al., 2021 [60]	Thoracic	Rat	Implant	Injectable collagen hydrogel	NA	+	+	NA	+
Silva NA, et al., 2010 [59]	Thoracic	Rat	Implant	Starch/poly-e-caprolactone blend and gellan gum	+	NA	NA	NA	NA
Suzuki H, et al., 2015 [61]	Thoracic	Rat	Implant	Collagen filaments	NA	+	+	NA	NA
Yara T, et al., 2009 [62]	Thoracic	Rat	Implant	Collagen filaments	NA	+	+	NA	NA

Note: -, no difference with the control group; +, effective; NA, not available.

**Table 2:** Summary of the included studies and the effect of biodegradable scaffolds application in SCI.

					Effect On Pathophysiological Events				
Author, Year	Location of Injury	Species	Application	Biodegradable Scaffold/(Character)	Anti-Inflammation	Scar/Cavity	Axon Growth	Angiogenesis	Motor Functional Recovery
Kubinová Š, et al., 2015 [66]	Thoracic	Rat	Implant	SIKVAV-modified PHEMA	NA	+	+	+	NA
Hejcl A, et al., 2008 [67]	Thoracic	Rat	Implant	2-hydroxyethyl methacrylate	NA	+	+	+	NA
Slotkin JR, et al., 2017 [69]	Thoracic	The green monkey	Implant	Poly-lactic-co-glycolic acid and Poly-l-lysine	+	+	+	NA	NA
Silva NA, et al., 2013 [70]	Thoracic	Rat	Implant	Starch with polycaprolactone	NA	+	+	NA	+
Thomas AM, et al., 2013 [71]	Thoracic	Rat/Mouse	Implant	Poly(lactide-co-glycolide) multiple channel bridges	NA	+	+	NA	NA
Man W, et al., 2021 [72]	Thoracic	Rat	Implant	Hierarchically aligned fibrin hydrogel and functionalized self-assembling peptides	NA	+	+	+	+
Kubinová S, et al., 2011 [73]	Thoracic	Rat	Implant	Highly superporous cholesterol-modified poly(2-hydroxyethyl methacrylate) scaffolds	NA	+	+	+	NA
Guest JD, et al., 2018 [74]	Thoracic	Thoracic	Implant	PLGA-PLL	NA	+	+	NA	-
Hakim JS, et al., 2019 [75]	Thoracic	Rat	Implant	PLGA-PLL	NA	+	+	NA	-
Anzalone A, et al., 2018 [76]	Cervical	Mouse	Implant	Poly-lactic-co-glycolic	NA	NA	+	NA	NA
De Laporte L, et al., 2009 [77]	Thoracic	Rat	Implant	Poly-lactic-co-glycolic/(Lipoplex incubation on ECM-coated PLG)	NA	NA	NA	NA	NA
Wong DY, et al., 2008 [78]	Thoracic	Rat	Implant	Salt-leached porous poly (epsilon-caprolactone)	NA	NA	+	NA	NA
Ribeiro-Samy S, et al., 2013 [79]	Thoracic	Rat	Implant	Poly(3-hydroxybutyrateco- 3-hydroxyvalerate) (PHB-HV)	NA	NA	NA	NA	-
Pawar K, et al., 2015 [80]	Cervical	Mouse	Implant	Poly-lactic-co-glycolic	NA	NA	+	NA	+
Rooney GE, et al., 2008 [81]	Thoracic	Rat	Implant	Radiopaque barium sulfate-impregnated poly-lactic-co-glycolic acid	NA	NA	NA	NA	NA
Shu B, et al., 2019 [82]	Thoracic	Rat	Implant	PLA-PPy	+	+	+	NA	+
Zhou L, et al., 2018 [83]	Thoracic	Mouse	Implant	Plant-derived polyphenol, tannic acid (TA), cross-linking and doping conducting polypyrrole (PPy) chains	NA	+	+	NA	+
Pertici VA, et al., 2014 [84]	Thoracic	Rat	Implant	PLA-b-PHEMA block copolymer	+	+	+	+	+
Reis KP, et al., 2020 [85]	Thoracic	Rat	Implant	Valproic acid (VPA)/PLGA (Microfiber)	+	+	+	+	+
Novikova LN, et al., 2017 [86]	Cervical	Rat	Implant	Trimethylene carbonate and e-caprolactone (TC) containing poly-p-dioxanone microfilaments (PDO)	-	+	+	NA	NA

Note: -, no difference with the control group; +, effective; NA, not available.

**Table 3:** Summary of the included studies and the effect of nano-/micro-scale biomaterial scaffolds application in SCI.

					Effect on Pathophysiological Events				
Author, Year	Location of Injury	Species	Application	Nanomaterial Scaffold/Material	Anti-Inflammation	Scar/Cavity	Axon Growth	Angiogenesis	Motor Functional Recovery
Zamani F, et al., 2014 [89]	Thoracic	Rat	Implant	3D nanofibrous core–sheath scaffold/PLGA	NA	NA	+	+	+
Sun X, et al., 2019 [90]	Thoracic	Rat	Implant	Nano-fibrous channel wall/PLLA	+	+	+	NA	+
Cigognini D, et al., 2014 [91]	Thoracic	Rat	Injected	Nanostructures of two self-assembling peptides B24 and biotin-LDLK12	NA	NA	+	NA	NA
Yao S, et al., 2018 [92]	Thoracic	Rat	Implant	Hierarchically aligned fibrin nanofiber/Fibrin hydrogel	NA	+	+	+	+
Altinova H, et al., 2016 [93]	Cervical	Rat	Implant	Microstructured scaffold/Collagen	+	+	+	+	+
Usmani S, et al., 2020 [94]	Thoracic	Rat	Implant	Artificial nanotube/Carbon	+	+	+	NA	+
Sever-Bahcekapili M, et al., 2020 [95]	Thoracic	Rat	Implant	Neuroactive peptide nanofibers/ LN-PA, GAG-PA	NA	+	+	NA	+
Zhao T, et al., 2018 [96]	Thoracic	Rat	Implant	Nanofibrous scaffolds/ PHBV, PLA, Collagen	NA	+	+	NA	-
Chedly JL, et al., 2017 [97]	Thoracic	Rat	Implant	Microhydrogel scaffold/Chitosan	+	+	+	+	+
Cigognini D, et al., 2011 [98]	Thoracic	Rat	Implant	Nanomaterial SAPs with bone marrow homing motif (BMHP1)	+	+	+	+	+
Palejwala AH, et al., 2016 [99]	Thoracic	Rat	Implant	Poly (3-hydroxybutyrateco- 3-hydroxyvalerate) (PHB-HV)	NA	NA	NA	NA	-
Palejwala AH, et al., 2016 [99]	Thoracic	Rat	Implant	Nanoscaffolds	NA	+	+	+	NA
Pawelec KM, et al., 2018 [100]	Thoracic	Rat	Implant	Microstructure multi-channel scaffold/PCL	NA	NA	+	NA	NA
Milbreta U, 2016 [101]	Cervical	Rat	Implant	3D nanofiber scaffold/Collagen	+	+	+	NA	NA
Tysseling VM, et al., 2010 [102]	Thoracic	Rat	Injected	Peptide amphiphile (PA) molecules that self-assemble and display the laminin epitope IKVAV	NA	+	+	NA	+
Liu Y, et al., 2013 [103]	Thoracic	Rat	Injected	A self-assembling peptide/ K2(QL)6K2 (QL6)	+	+	+	NA	+

Note: -, no difference with the control group; +, effective; NA, not available.

**Table 4:** Summary of the included studies and the effect of biomaterial scaffolds in combinatory treatment for SCI.

					Effect on Pathophysiological Events					
Author, Year	Location of Injury	Species	Combinatory Agent	Biomaterial Scaffold	Anti-Inflammation	Scar/Cavity	Axon Growth	Angiogenesis	Facilitation of Cell Migration	Motor Functional Recovery
Furuya T, et al., 2013 [105]	Thoracic	Rat	bFGF	Gelatin hydrogel	NA	NA	NA	NA	NA	NA
Chantal SA, et al., 2008 [106]	Thoracic	Rat	Methylprednisolone	Biodegradable PLGA-based nanoparticles	+	+	NA	NA	+	NA
Jain A, et al., 2011 [108]	Thoracic	Rat	Constitutively active Cdc42, Rac1, BDNF	Microtubule-mediated slow release of BDNF	+	+	+	NA	+	NA
Wen Y et al., 2016 [109]	Thoracic	Rat	Anti-Nogo receptor antibody	PLGA microspheres containing BDNF and VEGF	+	+	+	+	+	+
Chen B, et al., 2015 [110]	Thoracic	Rat	bFGF	HEMA-MOETACL hydrogel	NA	+	+	NA	NA	+
Lin J, et al., 2019 [114]	Thoracic	Rat	Rehabilitation	Hybrid fiber-hydrogel scaffold	+	+	+	NA	+	+
Shi Q, et al., 2014 [115]	Thoracic	Rat	bFGF	Collagen scaffold	NA	+	+	NA	+	+
Wang X, et al., 2013 [116]	Thoracic	Rat	NT-3	Chitosan-based tube scaffold	NA	+	+	NA	+	+
Li G, et al., 2016 [117]	Thoracic	Rat and canine	NT-3	Fibrin-coated gelatin sponge scaffold	+	+	+	NA	+	+
Wei YT, et al., 2010 [118]	Thoracic	Rat	Nogo-66 receptor antibody	Hyaluronic acid -based hydrogels modified with poly-L-lysine (PLL)	+	+	+	+	+	NA
Bighinati A, et al., 2020 [119]	Thoracic	Rat	Ibuprofen and triiodothyronine	PLLA	+	+	+	NA	+	+
Ehsanipour A, et al., 2021 [120]	Thoracic	Mouse	BDNF	Hyaluronic acid (HA)-based, spherical microparticle	+	+	+	NA	+	+
Xie J, et al., 2022 [121]	Thoracic	Mouse	Sonic hedgehog (Shh) and retinoic acid (RA)	Magnesium oxide (MgO)/ poly (l-lactide-co-ε-caprolactone) (PLCL) scaffold	+	+	+	NA	+	NA
Xi K, et al., 2020 [122]	Thoracic	Rat	NGF	Microenvironment-responsive immunoregulatory electrospun fibers	+	+	+	NA	+	+
Rooney GE, et al., 2011 [123]	Thoracic	Rat	Dibutyryl cyclic adenosine monophosphate (dbcAMP)	Oligo [(polyethylene glycol) fumarate] (OPF) hydrogel scaffolds	NA	NA	+	NA	NA	NA
Stropkovská A, et al., 2022 [124]	Thoracic	Rat	Rho-A-kinase inhibitor	Chitosan/collagen porous scaffold	+	+	+	NA	+	NA
Man W, et al., 2021 [72]	Thoracic	Rat	Hierarchically aligned fibrin hydrogel	Functionalized self-assembling peptides (fSAP)	+	+	+	+	+	+
Smith DR, et al., 2020 [128]	Cervical	Mouse	IL-10 and NT-3	Multiple channel PLG	+	NA	+	NA	+	+
Breen BA, et al., 2017 [130]	Thoracic	Rat	NT-3	Injectable collagen scaffold	NA	+	+	NA	+	+
Wen Y et al., 2016 [109]	Thoracic	Rat	AntiNogo, BDNF and vascular endothelial growth factor	Hyaluronic acid (HA) hydrogel	+	+	+	+	+	+
Jain A, et al., 2006 [129]	Thoracic	Rat	BDNF	Gelling agarose hydrogels	NA	+	+	NA	+	NA

Note: +, effective; NA, not available.

**Table 5:** Summary of the included studies and the effect of combinatory cell therapy for SCI.

Type of Grafted Cells	Biomaterial Scaffold	Results/Advantages	Limitations/Disadvantages
NPCs	PLGA scaffold HA scaffold Protein-functionalized chitosan scaffold 3D biomimetic hydroge Collagen microchannel scaffold 3D printed heparin sulfate-collagen scaffold Exosomes-collagen scaffold Multi-channel collagen scaffold Aligned collagen scaffold Polymer scaffold Chitosan channels scaffold Protein-functionalized chitosan scaffold Laminin-coated pHEMA-MOETACl Hydrogel Artificial microfiber scaffold Polycaprolactone electrospun fiber scaffold Fibrin scaffold SAP scaffold Matrigel scaffold	Functional recovery Graft cells survival and neuronal cell differentiation Secretion of trophic factors Protection of host neuronal cells Axonal outgrowth through injured lesion Remyelination of host axons Neuronal differentiation Host cells survival	Immune rejection Tumorgenesis
BMCSs	Chitosan-based thermosensitive scaffold Chitosan conduits scaffold Alginate hydrogel biomaterial PLGA scaffold Collagen scaffold Collagen filaments scaffold Porous collagen scaffold NeuroRegen scaffold PLGA scaffold HA-PLL scaffold SAP hydrogel scaffold Biologic scaffolds derived from fibrin and blood plasma Goldnanoparticles (Au NPs)-loaded Agarose/Poly (N- isopropylacrylamide) (PNIPAM) Thermosensitive quaternary ammonium chloride chitosan/β-glycerophosphate (HACC/β-GP) hydrogel scaffold Gelatin sponge scaffold Nanofibrous silk scaffold Cylindrical poly(D,L-lactide-co-glycolide)/small intestinal submucosa scaffold PHEMA scaffold	Functional recovery Repair of spinal cord injury Secretion of trophic factors Protection of host neuronal cells Axonal outgrowth Remyelination of host axons Host cells survival Low risk of immune rejection Autologous transplants No ethical issues	Difficulty of neuronal differentiation Low cell survival rate
Umbilical MSCs/Wharton’s jelly-derived MSCs	Collagen scaffold 3D printed collagen/silk fibroin scaffold HA-PH modified with the integrin-binding peptide arginine-glycine-aspartic acid scaffold		
Adipose-derived stem/stromal cells	Silk fibroin/chitosan scaffold Matrigel scaffold Resorbable poly(α-hydroxyacid) guidance channels scaffold		
Schwann cells (SCs)	Biodegradable poly-b-hydroxybutyrate scaffold Oligo[poly(ethylene glycol) fumarate] scaffold	Axon growth into SCs implants Ensheathment and myelination No tumorigenicity Modest but significant motor and sensory improvement SCs-elicited responses such as survivability post-transplantation, axon growth, and functional recovery can be improved with appropriate combination treatments Remyelination Functional recovery Secretion of trophic factors	No differentiation into neurons and astrocytes
OECs	PLGA Scaffold	Functional recovery Promotion of cell differentiation Inhibition of astrocyte formation Accelerate neuronal regeneration Secrete nerve growth factors Decrease neuronal apoptosis Reduce glial scaring Produce a number of trophic factors such as VEGF Constitute the myelin and the Ranvier nodes of the axons	No differentiation into neurons and astrocytes
Spinal cord-derived ependymal progenitor cells	HA containing PLA fibers scaffold	Preserve the neuronal tissue Diminish astrocytic reactivity surrounding the scar area Axonal outgrowth	No functional recovery
Dental pulp stem cells/Dental follicle cells	Chitosan scaffolds Aligned electrospun PCL/PLGA material scaffold	Increase the levels of BDNF, GDNF, beta-NGF and NT-3 Recovery of hind limb locomotor functions	No differentiation into neurons and astrocytes
NPCs and Schwann cells	3D bioprinting of NSC-laden HBC/HA/MA scaffold PLGA scaffolds Biodegradable polymer scaffold Poly (L-lactic-co-glycolic acid) scaffold	Provision of an ideal microenvironment for the growth and neural differentiation of grafted cells. Restoration of locomotor function Simulation of the parallel linear structure of spinal cord for optimal neuron regeneration and connection.	Immune rejection Tumorgenesis
BMSCs and Schwann cells	Multichannel polymer scaffold	Functional recovery Secretion of trophic factors Protection of host neuronal cells Axonal outgrowth through injured lesion Remyelination of host axons	No differentiation into neurons and astrocytes
Endometrial stem cells and Schwann cells	Degradable polymer implant PCL/gelatin nanofibrous scaffold	Functional recovery Secretion of trophic factors Protection of host neuronal cells Axonal outgrowth through injured lesion Remyelination of host axons Host cells survival	No differentiation into neurons and astrocytes
Dermal fibroblast-reprogrammed neurons	3D silk fibrous material	Functional recovery Axonal outgrowth through injured lesion Remyelination of host axons	No differentiation into neurons and astrocytes
Adipose-derived stem cells and OECs	Serum-derived albumin scaffold	Functional recovery Secretion of trophic factors Protection of host neuronal cells Axonal outgrowth through injured lesion Remyelination of host axons	No differentiation into neurons and astrocytes
NPCs and MSCs	3D longitudinal scaffold	Functional recovery Graft cells survival and neuronal cell differentiation Secretion of trophic factors Protection of host neuronal cells Axonal outgrowth through injured lesion Remyelination of host axons Neuronal differentiation Host cells survival	Immune rejection Tumorgenesis

**Table 6:** Biomaterial scaffolds in clinical trials for SCI (https://www.clinicaltrials.gov/, accessed on 1 August 2022).

								Effect on Pathophysiological Events				
Author, Year	Location of Injury	Ongoing Clinical Trials (Identifier)	Phase	Combinatory Agent	Biomaterial Scaffold	Motor Function	Sensory Function	Anti-Inflammation	Scar/Cavity	Axon Growth	Angiogenesis	Facilitation of Cell Migration
-	Cervical/Thoracic	NCT02688049	Phase 1 Phase 2	NSCs and MSCs	NeuroRegen scaffold	-	-	-	-	-	-	-
-	Cervical/Thoracic	NCT02352077	Phase 1	Bone marrow mononuclear cells and MSCs	NeuroRegen scaffold	-	-	-	-	-	-	-
-	Cervical/Thoracic	NCT02688062	Phase 1 Phase 2	Bone marrow mononuclear cells	NeuroRegen scaffold	-	-	-	-	-	-	-
-	Thoracic	NCT02138110	Not Applicable		Poly(lactic-co-glycolic acid)-b-poly(L-lysine) scaffold	-	-	-	-	-	-	-
-	Thoracic	NCT03762655	Not Applicable		Poly(lactic-co-glycolic acid)-b-poly(L-lysine) scaffold	-	-	-	-	-	-	-
-	Thoracic	NCT02510365	Phase 1		Collagen scaffold	-	-	-	-	-	-	-
-	Cervical/Thoracic	NCT03966794	Phase 1 Phase 2	Epidural Electrical Stimulation	Collagen scaffold	-	-	-	-	-	-	-
-	Thoracic/Lumbar	NCT02326662	Phase 1 Phase 2	Autologous NSCs	RMx Biomatrix	-	-	-	-	-	-	-
Amr SM, et al., 2014 [226]	Thoracic	-	-	BMSCs/peripheral nerve grafts	Chitosan-laminin scaffold	Several cases improved	Several cases improved	NA	NA	NA	NA	NA
Xiao Z, et al., 2018 [237]	Cervical/Thoracic	-	-	MSCs	NeuroRegen scaffold	Several cases improved	Several cases improved	NA	NA	NA	NA	NA
Chen W, et al., 2020 [133]	Thoracic	-	-	Bone marrow mononuclear cells	NeuroRegen scaffold	-	Several cases improved	NA	NA	NA	NA	NA

Note: -, no difference with the control group; NA, not available.

## Data Availability

Not applicable.
